# Can pain influence the proprioception and the motor behavior in subjects with mild and moderate knee osteoarthritis?

**DOI:** 10.1186/1471-2474-15-321

**Published:** 2014-09-27

**Authors:** Daniela C Silveira de Oliveira, Saulo Delfino Barboza, Franciele Dias da Costa, Monnique Ponciano Cabral, Vanessa Martins Pereira Silva, Valdeci Carlos Dionisio

**Affiliations:** Master Program in Health Sciences, Faculty of Medicine, Federal University of Uberlândia, Uberlândia, Minas Gerais Brazil; Physical Therapy Course, Faculty of Physical Education, Federal University of Uberlândia, Uberlândia, Minas Gerais Brazil; Mailing Address: Rua Benjamin Constant, 1286, Aparecida, Uberlândia, Minas Gerais Brazil

**Keywords:** *Knee* osteoarthritis, WOMAC, Pain, Proprioception, Electromyography

## Abstract

**Background:**

Osteoarthritis (OA) is a chronic disease, usually characterized by pain, which is associated with reduced muscle strength, disability and progressive loss of function. However, the pain influence over proprioception and motor behaviour remains unclear. Thus, the purpose of the study was to identify the levels of pain, the proprioceptive acuity and the pattern of muscle recruitment during stair ascent and descent in elderly patients with mild and moderate osteoarthritis (OA) compared to healthy subjects.

**Methods:**

The study participants included 11 healthy elderly subjects (7 women and 4 men) and 31 elderly patients with knee OA (19 women and 12 men). The functional capacity was assessed by the Western Ontario and McMaster Universities (WOMAC) osteoarthritis index; the pain was evaluated by Wong-Baker faces pain rating scale (WBS) and pressure pain threshold (PPT); the proprioceptive acuity was based on the joint position sense evaluated by electrogoniometer; and the electromyographic (EMG) activity of the major muscles of the lower limb were evaluated during a task of stair ascent and descent of 15 cm. For statistical analysis it was used Statistic for Windows software (StatSoft Inc., version 5.0). Data from the WOMAC index, WBS, the proprioceptive acuity and IEMG (for each muscle in each phase) were analyzed using the Mann–Whitney *U* test and data from PPT was used Kruskal-Wallis test.

**Results:**

Higher scores were found in the WOMAC index and WBS whereas lower scores were seen in PPT in patients with knee OA compared to healthy subjects. In contrast, there were no significant differences in the proprioceptive acuity and EMG results of most muscles analyzed between the groups.

**Conclusion:**

The presence of pain does not influence the proprioception and the motor behavior of the thigh muscles during stair ascent and descent in subjects with mild and moderate knee OA.

**Electronic supplementary material:**

The online version of this article (doi:10.1186/1471-2474-15-321) contains supplementary material, which is available to authorized users.

## Background

Osteoarthritis (OA) is a chronic disease that is associated with reduced muscle strength, disability and progressive loss of function, such as walking, climbing up and down stairs, and other tasks of the lower limbs, being the knee joint the most affected. Furthermore, the OA is usually related to pain [1–3].

The weakness of the quadriceps femoris muscle (QF) is a common finding in patients with OA, symptomatic or not [2]. In addition to disuse because of pain, it has also been reported the arthrogenic muscle inhibition (AMI) in OA [4], which along with weakness of the QF muscle, would be linked to inflammation, pain, joint laxity and damaged structures.

The pain may be local, but may also affect distant areas of the knee [5] and become chronic in patients with OA [6]. The progression from acute to chronic nociception is not a fully understood process. Researches in humans [7] and animals [8] suggest that peripheral mechanisms in the acute pain and intense nociceptive stimuli on the posterior column of the spinal cord can facilitate the transition from acute to chronic process through metabolic changes and a medullar reorganization [6–8]. Imamura et al. [9] identified clinically widespread hyperalgesia (superficial and deep structures) in disabled subjects due to late knee OA, suggesting that the central and peripheral nervous systems may be involved in the maintenance of chronic pain. Therefore the AMI would be generated by changes in the sensory receptors triggering due to damages in the knee joint and changes in the excitability of spinal pathways [4], which could affect the performance of functional activities.

Kinematic and kinetic studies showed that a greater range of motion (ROM) of the knee is required to climb stairs compared with the level of walking [10]. For the subjects with OA, climbing up and down stairs are more difficult and demanding tasks than walking [3, 11, 12]. However, these subjects may use different movements and patterns of muscle activation, possibly related to pain, when walking and climbing stairs compared to healthy subjects [13–15]. These differences in the movement and kinematic patterns may also be associated with proprioception, since it can influence the movement patterns [16] and motor control. The impaired proprioception may contribute to functional debility [17], reduced accuracy of leg movement [18] and is related to muscle weakness [19].

Here we hypothesized that pain could contribute to proprioceptive deficits and changes in the pattern of muscle recruitment during a task of climbing up and down stairs in subjects with mild and moderate OA. Thus, the aim of this study was to identify the levels of pain, the proprioception and the pattern of muscle recruitment during functional activities, such as climbing up and down stairs in elderly subjects with mild and moderate knee OA compared to healthy subjects.

## Methods

### Subjects

The study participants included 11 healthy elderly subjects (7 women and 4 men) and 31 elderly subjects with knee OA (19 women and 12 men). The anthropometric characteristics of the participants are shown in Table 1. The subjects were referred by rheumatologists.

**Table 1:** **Anthropometric characteristics of the study subjects**

	Knee OA (n = 31)	Healthy (n = 11)		
**Characteristics**	**Mean** **±** **SD**	**Mean** **±** **SD**	**t-value**	**p value**
Age (y)	60.5 ± 8.9	57.8 ± 6.2	0.9	0.3
Weight (Kg)	78.0 ± 13.5	71.7 ± 15.1	1.2	0.2
Height (m)	1.6 ± 0.1	1.7 ± 0.1	-0.9	0.3
BMI (kg/m^2^)	29.6 ± 4.5	25.9 ± 3.8	2.4	0.01^Ŧ^
Sex distribution	F: n = 11 UI and	F: n = 7; M: n = 4.	— — — —	— — — —
	n = 7 BI;			
	M: n = 5 UI and			
	n = 8 BI.			

OA: osteoarthritis; BMI: Body Mass Index; F: female; M: male; UI: unilateral involvement; BI: bilateral involvement ; ^Ŧ^Statistical significance.

As inclusion criteria for study participation the subjects should be 50 years old or more, presenting knee pain for six months or more, with diagnosis of OA according to the criteria of the American College of Rheumatology [20]. The diagnosis was supported by radiological evidence, with mild or moderate alteration of one or more compartments of the knee, with unilateral or bilateral involvement. The subjects could not present other musculoskeletal disorders, chronic inflammatory diseases as autoimmune diseases (rheumatoid arthritis, lupus, and gout), diabetes mellitus, and neuromuscular disorders as Parkinson’s disease, vertigo and other conditions that could affect the sensory capacity and movement control.

Before the start of data collection, the selected subjects were informed about the study and signed an informed consent approved by the Institutional Ethics Committee in Research under number 0012/2010. For all tests, the limb involved or more involved was evaluated.

### Assessment of functional quality

The assessment of pain and physical function of the knee was performed using the Western Ontario and McMaster Universities (WOMAC) osteoarthritis index [21], which outlines the domains of pain, joint stiffness and function.

### Pain assessment

A digital force gauge (Force TEN™ FDX, Wagner Instruments, Greenwich, CT, USA) with flat head of ½ inch in diameter was used for the assessment of superficial and deep pressure pain threshold (PPT) in the knee region [9]. The measurements were performed in myotomes related to the knee (rectus femoris - RF, vastus lateralis - VL, and vastus medialis - VM) and sclerotomes (pes anserinus bursae and patellar tendon). The PPT was expressed in kilograms force (Kgf), with higher values meaning less severe symptoms.

Pain was also assessed by pain scale with 6 faces (Wong-Baker faces pain rating scale - WBS), where the patient marked the face that better described the pain intensity. This scale is numbered from 0 to 5 points [22].

### Evaluation of proprioception

The proprioceptive acuity is based on the joint position sense, which was evaluated according Felson et al. [23]. For this purpose, subjects were asked to sit in a chair, with hips and knees flexed at 90° and trunk supported. The electrogoniometer was placed on the knee joint, considering the lateral epicondyle of the femur as reference. The subjects were instructed to extend the knee up to find the hand of the investigator (test position) and kept it in this position for 5 seconds. Next, the subjects were asked to return to the initial position of rest, thus remaining for 3 seconds, and then repeat the previous movement, without the presence of the hand of the investigator (playback position) for another 5 seconds. At this time, the subjects were blindfolded and ears covered, and the movement was conducted in 10 different positions. For each repetition, the proprioceptive acuity was considered as the difference between the angles of the knee recorded from the display unit in the test and playback positions [23].

### Electromyographic and kinematic evaluation

After shaving and cleaning the skin with alcohol, active bipolar surface electrodes (DataHominis Tecnologia Ltda, Uberlândia, Brazil) were fixed on the gastrocnemius lateralis (GL), soleus (SO), tibialis anterior (TA), vastus medialis oblique (VMO), vastus medialis longus (VML), vastus lateralis (VL), and biceps femoris (BF) muscles. For the VMO muscle, the surface electrode was placed on the belly muscle, following the direction of the fibers [24]. For the other muscles, the electrodes were placed according to the guidelines of the Surface Electromyography for the Non-Invasive Assessment of Muscles (SENIAM) project [25]. Each electrode consisted of two parallel silver plates (1 cm long × 1 mm wide), placed 1 cm apart. The electrodes had magnification of 20 times, impedance of 10 GΩ and rejection of 92 dB, and were connected to a computerized device of electromyography (DataHominis Tecnologia Ltda), with a magnification of 100 times (2000 amplification of total time), a pass filter of 15 Hz to 1 kHz and an acquisition frequency of 2000 Hz.

For movement analysis, it was used an electrogoniometer (EMGsystem) with flexible poles and 270° rotation, which was placed on the knee joint (lateral epicondyle of the femur). Before starting the evaluation, the channels of the electrogoniometer were calibrated to determine the maximum range of 180°. It was determined 0° for the full knee extension and any value greater than 0° for the knee flexion.

### Procedures

After the subjects were submitted to the WOMAC questionnaire, pain and proprioception assessments, they were evaluated by means of electromyography (EMG) recordings during a task of climbing up and down a stair of 15 cm. For this purpose, the subjects were dressed in shorts and shirt, and were instructed to remain standing comfortably in front of the ladder and keep their arms close to the body with the head elevated in order to look forward. To ascent the stair the subjects used first the foot of the evaluated member. To descend from the stair the subjects received the same guidelines, but the non-evaluated member started the downward movement. For each movement, the subjects had a verbal command and seven replicates of each movement (rise or fall) were performed.

### Data analysis

The mean values of EMG activity and kinematics (displacement and angular velocity) were calculated for each of the tasks in the two groups. EMG and kinematic signals were processed offline using the Excel software and KaleidaGraph (rectification, low-pass filter at 25 Hz, normalization of data by the peak of each EMG activity, integral in different phases of the movement – IEMG). These values were calculated for three different phases, based on the angular knee displacement: 300 milliseconds before initiating the movement (P1), from P1 to the knee flexion peak (P2), and from P2 up to the total knee extension (P3).

For statistical analysis it was used Statistic for Windows software (StatSoft Inc., version 5.0). Data from the WOMAC index, WBS, the proprioceptive acuity and IEMG (for each muscle in each phase) were analyzed using the Mann–Whitney *U* test and data from PPT was used Kruskal-Wallis test. Values of p ≤ 0.05 (α error) were considered statistically significant.

## Results and discussion

Significant differences were found between the groups in the parameters of pain, joint stiffness and function as assessed by the WOMAC index (p < 0.0001) as well as in the subjective pain perception as assessed by WBS (p < 0.0001) (Table 2). Also, PPT assays showed significant differences between the groups in individual values for each myotome and sclerotome (p < 0.05), with the control group presenting lower pain sensitivity, that is, it was more tolerant to pain. In addition, there was a tendency to have significantly higher values in the angular velocity of the knee during stair ascent, but not stair descent, in healthy subjects compared to patients with knee OA (p = 0.06) (Table 2).

**Table 2:** **WOMAC index, WBS, and PPT, and angular velocity of the knee**

Variables	Knee OA (n = 31)	Healthy (n = 11)	p value
	Median (25%-75%)	Median (25%-75%)	
**WOMAC**			
Pain	55 (45–60)	0 (0–10)	<0.0001^Ŧ^
Stiffness	50 (37.5-62.5)	0 (0–0)	0.0001^Ŧ^
Function	51.5 (45.6-7.3)	2.9 (0–7.3)	<0.0001^Ŧ^
**WBS**	3 (2–4)	0 (0–0)	<0.0001^Ŧ^
**PPT**			
**Myotomes**			
RF	5.4 (3.4-8.0)	10.5 (8.2-12.0)	0.0002^Ŧ^
VL	3.9 (2.6-5.8)	7.5 (6.5-8.7)	0.0020^Ŧ^
VM	4.0 (2.9-5.6)	6.7 (5.6-8.2)	0.0008^Ŧ^
**Sclerotomes**			
Pes anserinus bursae	2.7 (1.4-4.8)	7.4 (3.5-8.8)	0.0014^Ŧ^
Patellar tendon	5.6 (2.9-8.2)	12.8 (9.9-13.8)	0.0002^Ŧ^
**Angular velocity**			
Stair ascent	278.9 (227.5-317.4)	368.1 (269.1-423.4)	0.0600
Stair descent	162.4 (131.6-198.6)	157.3 (128.4-211.7)	0.8700

Median and 25%-75% interquartile range of the variables observed during stair ascent and descent in patients with knee osteoarthritis (OA) and healthy subjects. WOMAC: Western Ontario and McMaster Universities osteoarthritis index; WBS: Wong-Baker faces pain rating scale; PPT: pressure pain threshold; RF: rectus femoris; VL: vastus lateralis; VM: vastus medialis; ^Ŧ^Statistical significance.

**Figure 1:**
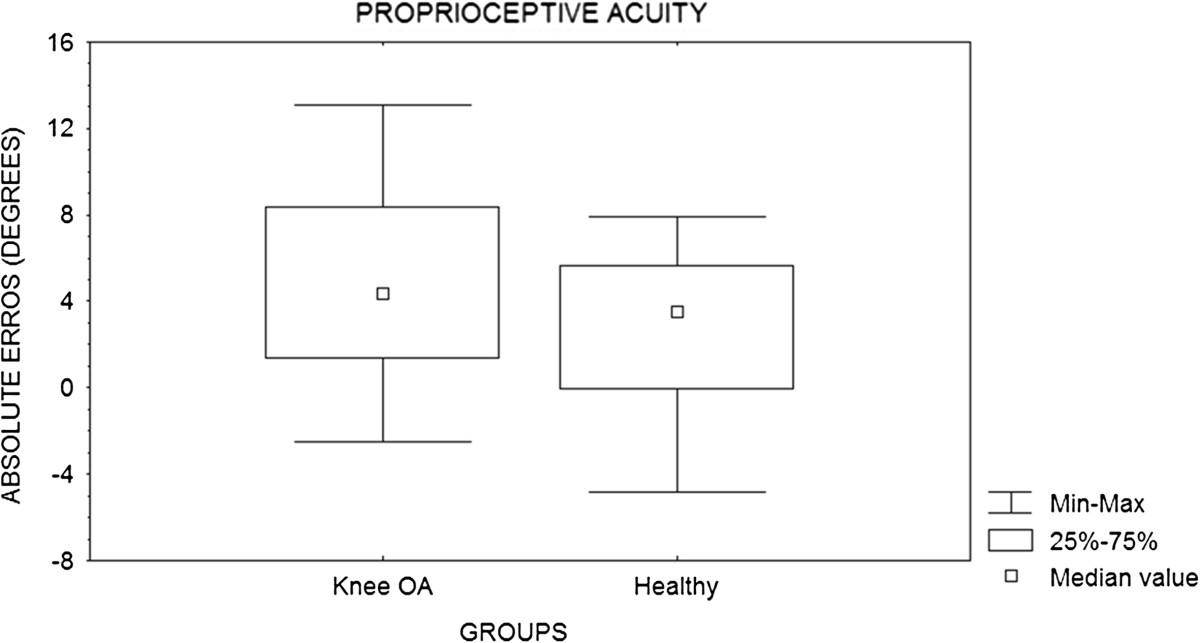
**Comparison of proprioception based on the joint position sense between the groups of patients with knee osteoarthritis (OA) and healthy subjects.**

**Figure 2:**
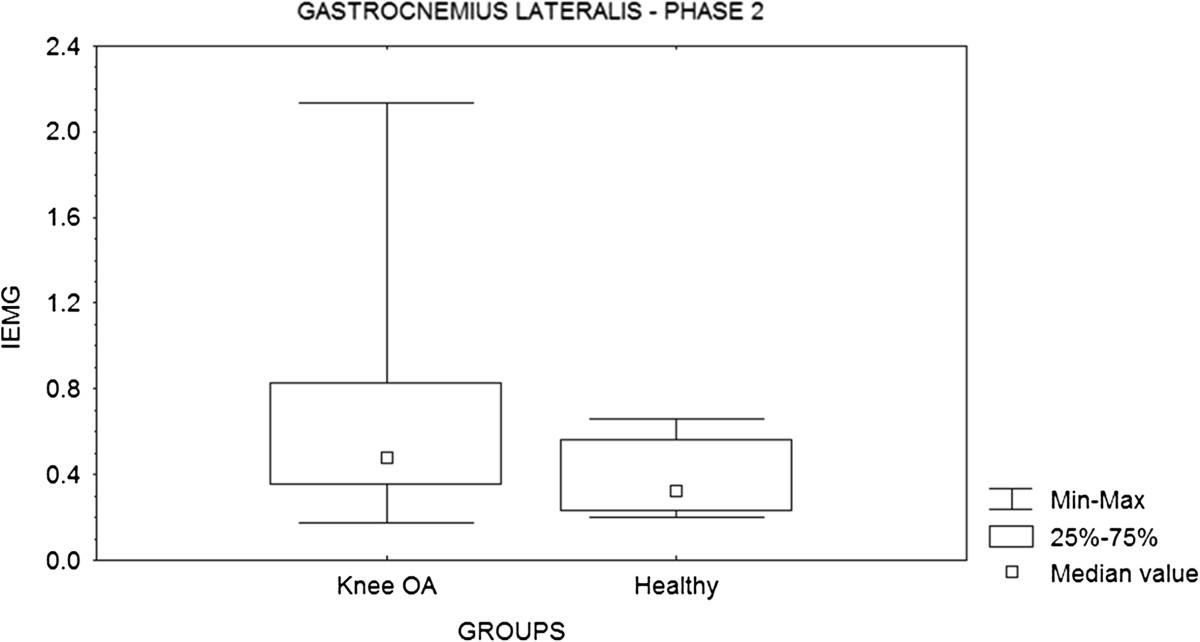
**Comparison between EMG values obtained from the gastrocnemius lateralis muscle of patients with knee osteoarthritis (OA) and healthy subjects during descent from a stair (phase 2).**

On the other hand, there were no significant differences (p = 0.23) between the groups in values obtained in proprioceptive acuity assays (Figure 1). Likewise, EMG results showed no significant differences in most muscles examined (Table 3), except for the GL muscle in phase 2 (p = 0.05) during stair descent (Figure 2).

**Table 3:** **Median and 25%-75% interquartile range of EMG activity for knee OA and healthy subjects**

	Stair ascent			Stair descent		
Muscles	Knee OA	Healthy	p value	Knee OA	Healthy	p value
	Median	Median		Median	Median	
	(25-75%)	(25-75%)		(25-75%)	(25-75%)	
GL^1*^	0.11	0.11	0.69	0.09	0.10	0.74
	(0.09-0.16)	(0.08-0.14)		(0.05-0.16)	(0.04-0.11)	
SO^1^	0.08	0.08	0.32	0.07	0.07	0.42
	(0.05-0.09)	(0.06-0.10)		(0.04-0.10)	(0.06-0.11)	
TA^1^	0.04	0.04	0.14	0.05	0.05	0.73
	(0.02-0.06)	(0.04-0.07)		(0.03-0.07)	(0.04-0.07)	
VMO^1^	0.02	0.02	0.98	0.03	0.03	0.57
	(0.01-0.03)	(0.01-0.04)		(0.02-0.04)	(0.02-0.06)	
VML^1^	0.06	0.05	0.76	0.08	0.06	0.85
	(0.04-0.08)	(0.04-0.08)		(0.05-0.11)	(0.04-0.12)	
VL^1^	0.06	0.04	0.39	0.06	0.06	0.68
	(0.03-0.08)	(0.03-0.06)		(0.04-0.09)	(0.03-0.08)	
BF^1^	0.07	0.10	0.06	0.10	0.12	0.37
	(0.05-0.11)	(0.08-0.13)		(0.06-0.13)	(0.06-0.14)	
GL^2^	0.20	0.16	0.11	0.48	0.32	0.05^Ŧ^
	(0.15-0.32)	(0.09-0.23)		(0.35-0.82)	(0.22-0.56)	
SO^2^	0.15	0.15	0.35	0.47	0.38	0.27
	(0.11-0.21)	(0.08-0.16)		(0.33-0.62)	(0.21-0.56)	
TA^2^	0.18	0.18	0.43	0.37	0.33	0.15
	(0.16-0.21)	(0.15-0.19)		(0.30-0.50)	(0.22-0.40)	
VMO^2^	0.06	0.04	0.34	0.30	0.28	0.28
	(0.03-0.08)	(0.02-0.07)		(0.25-0.38)	(0.20-0.34)	
VML^2^	0.12	0.09	0.11	0.49	0.38	0.10
	(0.09-0.16)	(0.07-0.11)		(0.35-0.65)	(0.27-0.42)	
VL^2^	0.12	0.07	0.10	0.40	0.31	0.22
	(0.06-0.19)	(0.04-0.11)		(0.29-0.57)	(0.26-0.38)	
BF^2^	0.16	0.15	0.67	0.53	0.37	0.32
	(0.10-0.22)	(0.11-0.27)		(0.35-0.64)	(0.30-0.67)	
GL^3^	0.61	0.57	0.43	0.80	0.70	0.57
	(0.52-1.06)	(0.48-0.87)		(0.48-1.17)	(0.56-1.07)	
SO^3^	0.54	0.58	0.87	0.73	0.79	0.77
	(0.39-0.76)	(0.47-0.63)		(0.56-0.81)	(0.55-0.87)	
TA^3^	0.38	0.34	0.38	0.24	0.19	0.29
	(0.26-0.62)	(0.24-0.43)		(0.14-0.33)	(0.15-0.21)	
VMO^3^	0.30	0.30	0.62	0.15	0.12	0.70
	(0.25-0.42)	(0.25-0.33)		(0.10-0.24)	(0.10-0.21)	
VML^3^	0.45	0.41	0.72	0.34	0.24	0.57
	(0.39-0.69)	(0.40-0.73)		(0.23-0.47)	(0.21-0.39)	
VL^3^	0.49	0.40	0.24	0.26	0.20	0.36
	(0.35-0.65)	(0.37-0.48)		(0.18-0.39)	(0.16-0.31)	
BF^3^	0.56	0.59	0.33	0.40	0.36	0.35
	(0.39-0.71)	(0.46-0.87)		(0.27-0.53)	(0.33-0.61)	

EMG activity of the gastrocnemius lateralis (GL), soleus (SO), tibialis anterior (TA), vastus medialis oblique (VMO), vastus medialis longus (VML), vastus lateralis (VL), and biceps femoris (BF) muscles of patients with knee osteoarthritis (OA) and healthy subjects during stair ascent and descent. *Superscript numbers in each muscle indicate the phase in which EMG signals were obtained; ^Ŧ^Statistical significance.

Our hypothesis was that pain could contribute to proprioceptive deficits and changes in the pattern of muscle recruitment during a task of climbing up and down stairs. The results do not support our hypothesis, since the pain did not cause a significant change in the pattern of muscle recruitment during a task of climbing up and down stairs, and no change in the proprioceptive acuity.

The higher body mass index (BMI) of the group with knee OA could influence pain, due to increased cartilage degeneration [26, 27] in function of substances that increase the inflammation and can contribute to joint damage [28, 29]. However, this difference represents values that approximate the level I of the obesity, and even then, did not influence the results, since the proprioceptive acuity and pattern of muscle recruitment were similar to the healthy group.

The presence of pain in patients with OA may have different mechanisms, like the mechanical tension on the nociceptors by local pressure or joint movement, irritation of nociceptors by inflammatory mediators [30], muscle contraction, QF weakness and joint effusion [4, 31]. The synovium, periosteum, subchondral bone, infrapatellar fat purse, and joint capsule are also structures that produce pain [28, 32]. The experimentally induced pain has indicated that it may have some influence on the proprioceptive system [33]. Chemicals substances produced in response to pain may sensitize free nerve endings, resulting in abnormal discharge of pain afferents. This could influence the motoneuron gamma, and consequently the activity of the muscle spindle, thus interfering with the proprioceptive entry [16]. Although pain is present in the subjects analyzed in our study, it had no influence on the proprioception. This suggests that proprioceptive acuity in subjects with knee OA may result from factors other than pain, like damages or reduction of the mechanoreceptors [31] and capsuloligamentous changes [16], like the ones in the OA more severe [34].

In our study, the participants with knee OA had mild or moderate levels and tissue was less damage than severe OA. For mild OA it was observed the lack of the correlation between muscle force and knee joint loading during gait [35]. This could also contribute not to support the hypothesis proposed by van der Esch et al. [19], which proprioception is related to muscle weakness and functional ability. The relationship between proprioceptive acuity and muscle force can be dependent on the level of severity of the OA and or pain.

More recently, research has begun to focus on other different mechanisms by which could lead to knee pain and they are classified into central and peripheral pathways [5]. Peripheral sensitization occurs when peripheral nociceptive afferents become spontaneously more active and cause local pain, so-called “primary hyperalgesia”. On the other hand, central sensitization is involved with referred pain and allodynia in a location away from the involved joint, with increased excitability and/or decreased inhibition at cortical or spinal level. The term “secondary hyperalgesia” refers to increased sensitivity of neurons in the posterior spinal cord in the corresponding segments to the primary site [36]. Likewise it was observed a lack of correlation between the radiological findings and pain [6]. The pain in OA also demonstrates an activation of the prefrontal cortex limbic region, suggesting that this region, also associated with emotional responses, may play a role in the generation of pain in OA [36]. Acute pain is usually self-limited and serves as a protective biological function, but chronic pain, in contrast, has no protective biological function, is not self-limiting, and can persist for years after the initial injury, and may also be more refractory to several modalities of treatment [6]. The participants of this study had at least six months pain history due to knee OA, suggesting a chronic pain and they might have developed a secondary hyperalgesia [31]. Thus, in the presence of chronic pain, subjects with knee OA could have other symptoms related to emotional issues, such as anxiety, difficulty in sleeping, depression [6], factors that were not analyzed in our study, but could have some kind of influence in the presence of pain in these subjects [37].

The biomechanical mechanisms or strategies could also justify the similarity in performing the task of climbing up and down stairs. The trunk could have been flexed, thus reducing the knee torque extensor [38, 39]. Fok et al. [39] observed that there is a greater anterior pelvic inclination and a smaller flexion angle, and lower knee extension time in both stair ascent and descent. This could be considered a limitation of the present study, since even though the subjects were instructed to remain upright, the trunk movements were not monitored, which may have contributed to our results.

Regarding the EMG results of the muscles analyzed, only the GL muscle of subjects with knee OA showed increased muscle activation during stair descent (phase 2) compared to healthy subjects. In the proposed task the assessed limb was the supporting limb, while the opposite limb reached the bottom stair. The increased activation of the GL muscle may be associated with an isometric contraction, since there are simultaneous knee flexion and ankle dorsiflexion, contributing for joints stabilization, which can cause control of the anterior displacement of the tibia. This observation is similar to the one observed in others studies during squats [24]. So, the GL facilitated slowing the movement of knee flexion, facilitating the execution of the activity. Likewise, Hortobágyi et al. [40] found that the rates of activation of the GL and TA muscles were significantly higher in subjects with OA than in healthy adults and young adults, with large variation between individuals. However, it is not possible to say that the pain has not influenced this activation.

Finally, another factor that may have influenced the muscle activation during stair ascent and descent in our study is that motor activity is time-dependent [15, 41, 42]. In this study a strong trend was observed for reducing velocity in stair ascent in subjects with knee OA. The reduction in gait velocity has been suggested as a possible method used by people with OA to reduce the loads on the knee [41, 42].

Although the self-selected velocity and no control of trunk movements can be considered as limitations of the study, the results are consistent with previous studies [6, 16, 31, 40]. Another possible limitation is the small sample, however, it is consistent with early studies [3, 15, 17]. The results of this study suggest that there is loss of association of pain with proprioceptive acuity and muscle activity during a functional task, which could be compensated by physiological and biomechanical mechanisms. This study also indicates that the pain in knee OA involves other physiological variables, which can lead to increased pain and functional decline.

## Conclusion

Although the pain is present in subjects with mild and moderate knee OA, it does not influence the proprioception and the motor behavior of the thigh muscles during stair ascent and descent.

## Authors’ original submitted files for images

Below are the links to the authors’ original submitted files for images. Authors’ original file for figure 1 Authors’ original file for figure 2

## Abbreviations

OA: Osteoarthtitis
AM: Arthrogenic muscle inhibition
QF: Quadriceps femoris
ROM: Range of motion
WOMAC: Western Ontario and McMaster Universities
PPT: Pressure pain threshold
RF: Rectus femoris
VL: Vastus lateralis
VM: Vastus medialis
Kgf: Kilograms force
WBS: Wong-Baker scale
GL: Gastrocnemius lateralis
SO: Soleus
TA: Tibialis anterior
VMO: Vastus medialis oblique
VML: Vastus medialis longus
BF: Biceps femoris
SENIAM: Surface Electromyography for the Non-Invasive Assessment of Muscles
EMG: Electromyography
IEMG: Integrated electromyography
BMI: Body mass index.
