# Supplementary figures and images for: Can pain influence the proprioception and the motor behavior in subjects with mild and moderate knee osteoarthritis?
Source: BMC Musculoskelet Disord. 2014 Sep 27;15:321. doi: 10.1186/1471-2474-15-321 (PMC4190294; doi:10.1186/1471-2474-15-321)

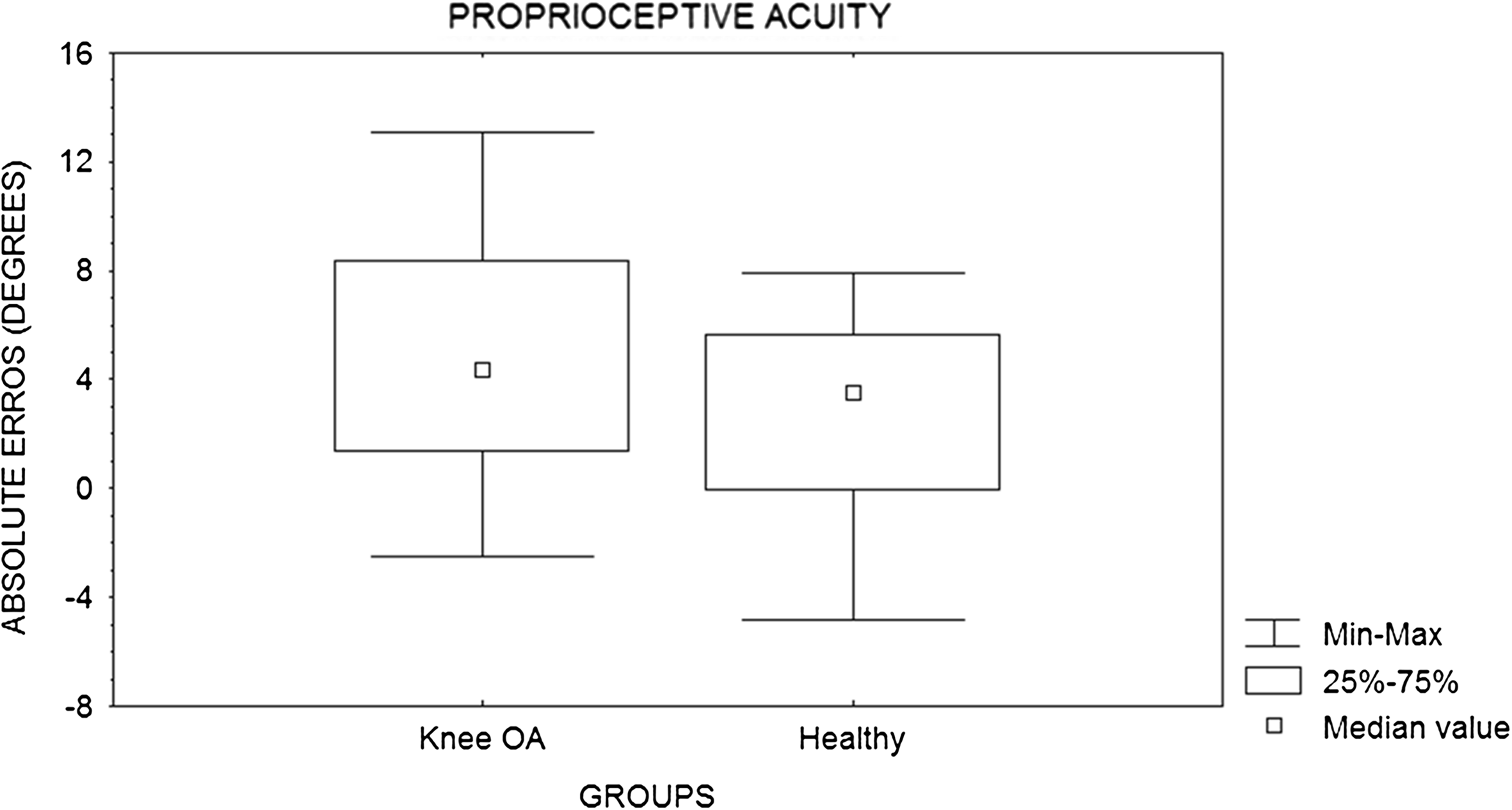

Supplement: Supplementary file 1 — Authors’ original file for figure 1 [file 12891_2014_2258_MOESM1_ESM.tiff]

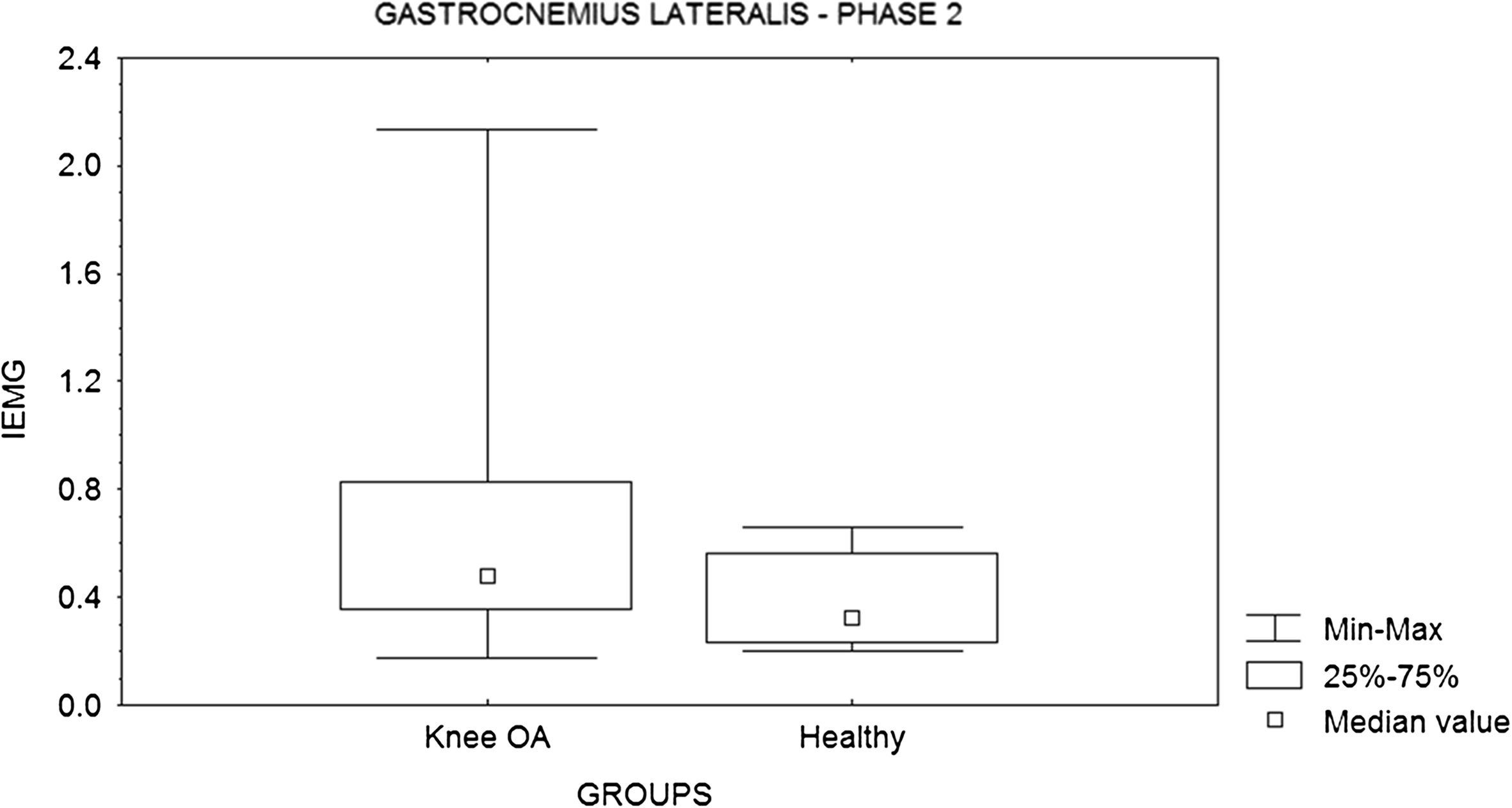

Supplement: Supplementary file 2 — Authors’ original file for figure 2 [file 12891_2014_2258_MOESM2_ESM.tiff]
